# Supplementary material for: Association between oral microbiome and seven types of cancers in East Asian population: a two-sample Mendelian randomization analysis
Source: Front Mol Biosci. 2023 Nov 21;10:1327893. doi: 10.3389/fmolb.2023.1327893 (PMC10702768; doi:10.3389/fmolb.2023.1327893)
Supplement: Supplementary file 1 [file DataSheet1.ZIP › Supplementary Materials/MR plots of saliva/saliva═╝/colorectal cancer/pheno.2020_to_colorectal cancer_leave_one_out.pdf]

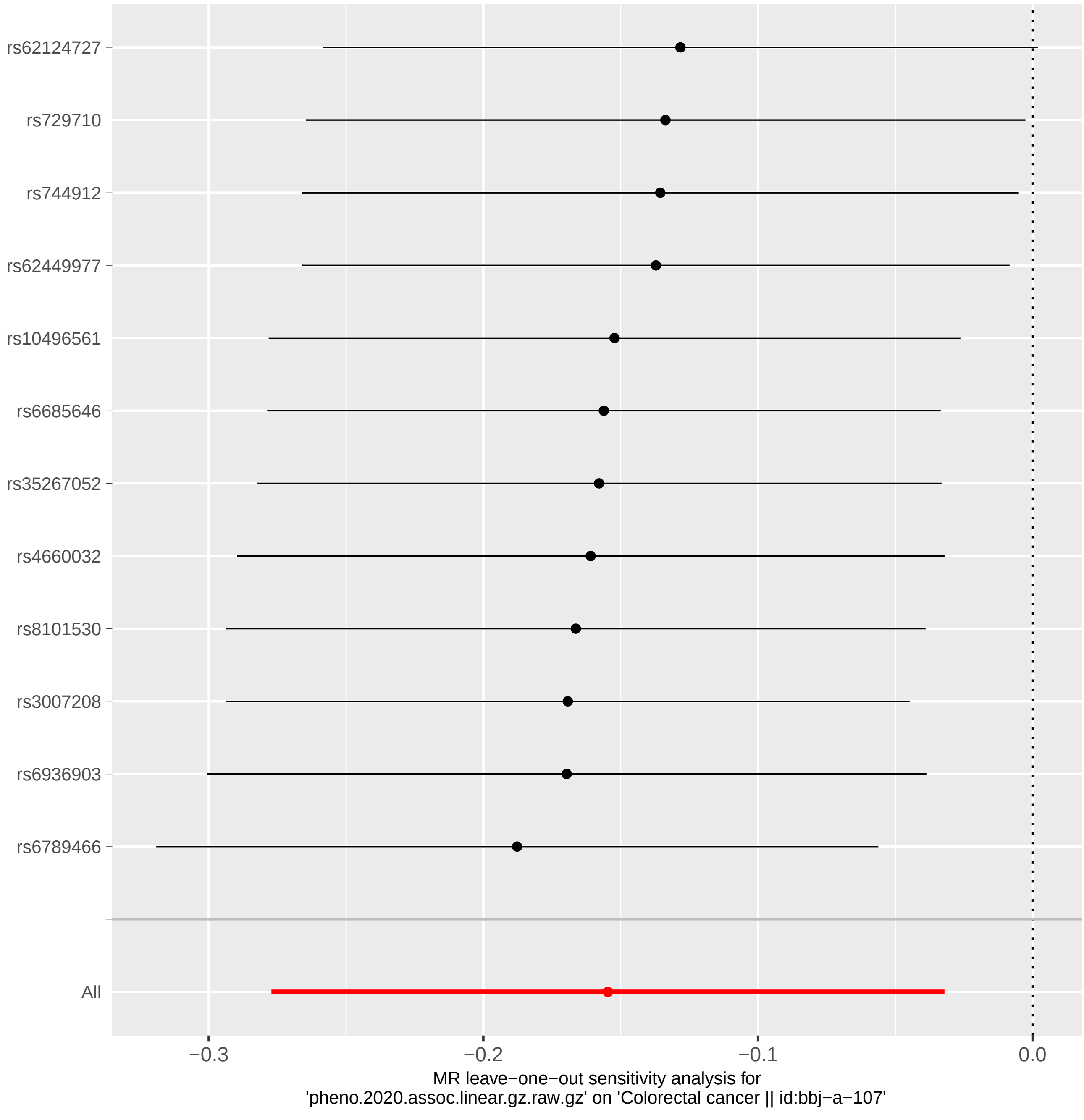
rs62124727
rs729710
rs744912
rs62449977
rs10496561
rs6685646
rs35267052
rs4660032
rs8101530
rs3007208
rs6936903
rs6789466
All
−0.3
−0.2
−0.1
0.0
MR leave−one−out sensitivity analysis for
'pheno.2020.assoc.linear.gz.raw.gz' on 'Colorectal cancer || id:bbj−a−107'
